# Postoperative stability following a triple pelvic osteotomy is affected by implant configuration: a finite element analysis

**DOI:** 10.1186/s13018-022-03169-3

**Published:** 2022-05-15

**Authors:** Henrik Hedelin, Erik Brynskog, Per Larnert, Johan Iraeus, Tero Laine, Kerstin Lagerstrand

**Affiliations:** 1 Department of Orthopaedics, Sahlgrenska University Hospital, Gothenburg, Sweden; 2 Department of Mechanics and Maritime Sciences, Chalmers University of Technology, Gothenburg, Sweden; 3 Institute of Clinical Sciences, Sahlgrenska Academy, University of Gothenburg, Gothenburg, Sweden; 4 Medical Physics and Biomedicine, Sahlgrenska University Hospital, Gothenburg, Sweden

**Keywords:** Osteotomy, Acetabulum, Fixation, Bioabsorbable screw, Finite element analysis

## Abstract

**Background:**

The triple pelvic osteotomy is an established surgical method with multiple modifications regarding surgical technique and choice of implant. The stability of the osteotomy is affected by numerous factors, and among these, the three-dimensional implant configuration is a scientifically less explored aspect.

**Methods:**

We used a finite element model of a hemi-pelvis with a standardized triple osteotomy to calculate relative flexibility for loads in all translational degrees of freedom for five different implant configurations. Two of the configurations used entry points only feasible when implant removal was not necessary.

**Results:**

The stability of the osteotomy improved with an increased distance between the implants in the plane of the osteotomy as well as for a more perpendicular angle relative to the osteotomy plane. The implant configurations with more entry points available made this easier to adhere to.

**Conclusion:**

The use of bioabsorbable implants may provide better opportunities for optimal implant constructs which can, to a certain degree, compensate for the lesser mechanical stiffness of bioabsorbable polymers as compared to metal implants.

**Supplementary Information:**

The online version contains supplementary material available at 10.1186/s13018-022-03169-3.

## Introduction

The triple pelvic osteotomy (TPO) is a re-directional osteotomy normally used in children over the age of 6–8 years old when the symphysis has become too stiff to allow a Salter osteotomy. The procedure is also an alternative to periacetabular osteotomies (PAO) in adults. Numerous modifications of the TPO have been proposed over the years with the Steel, Tönnis, and Carlioz modifications being the most well-known [1–3].

As in a Salter osteotomy, the TPO entails a cut of the ilium, and in addition also osteotomies of the ischial and pubic bone. Since the entire acetabulum is mobilized, the TPO is inherently more unstable than most other pelvic osteotomies. Numerous different methods of fixation have been used for the iliac osteotomy, including K-wires, Steinmann pins, and screws and plates [4–6]. Recently, bioabsorbable screws have been presented as an alternative, negating the need for implant removal [7]. In clinical practice, many surgeons still use smooth K-wires for fixation [8], while some advocate threaded wires to reduce the risk of migration [9]. Fully threaded screws of varying diameter [10, 11] would be the choice by many surgeons in older children, while plate and screws solutions are rarely used.

The research regarding pelvic osteotomies has mainly been focusing on modifications of the osteotomies or type of implant used rather than how the positioning of the implants affects the stability of the osteotomy. The classic papers presenting the surgical method of a TPO or Salter osteotomy go into little detail regarding the exact placement of the implants. Salter simply states that “a stout Kirschner wire is inserted across the osteotomy site, through the graft and into the distal fragment posterior to the acetabulum in order to prevent any subsequent shift of the graft or of the distal fragment” [12]. Tönnis suggests the following fixation for his triple osteotomy: “The fragment is stabilized with four Kirschner wires 2.5 mm in diameter drilled in from the iliac crest. The wires are inserted in a fan-shaped pattern so that they diverge into the lateral, medial, anterior, and posterior parts of the acetabulum.”[13]. Carlioz, in his original article from 1982, also uses K-wires and mentions that he occasionally places a retrograde K-wire from an inferior/anterior point toward the proximal ilium, but he advises against this as routine practice due to difficulty in implant removal and that it may impair hip flexion [3].

Traditionally, the implants have been inserted from the iliac crest, in or close to the harvest site of the bone graft. The use of bioabsorbable screws, negating the need for implant removal, enables the surgeon to place the screws from any location that can be reached from the surgical approach [14]. Even though this could, based on basic orthopedic principles, provide a more advantageous biomechanical fixation, this has to our knowledge not been scientifically explored. One of the most elaborate studies on the subject is Yassir et al*.* who performed a cadaver-based study comparing the stability of three different screw configurations for a Tönnis triple osteotomy and a PAO [15]. In this study, the different screw configurations in the iliac osteotomy did not, to a great extent, affect stability. The setup did not, however, include a graft and only examined weight-bearing loads and the results can therefore not be expected to be representative for a postoperative clinical setting.

**Fig. 1:**
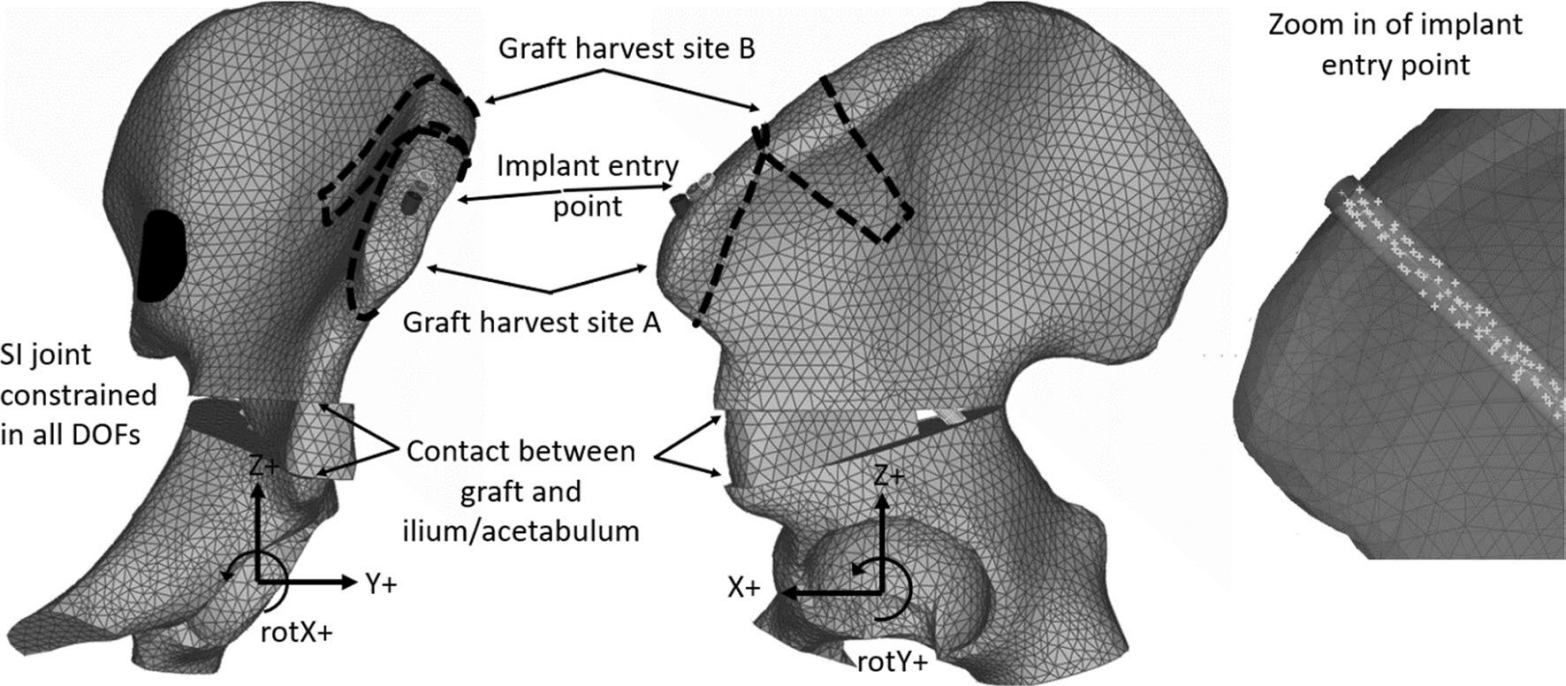
The base FE model of the pelvis. The pelvis is positioned in the standing anatomical position. A right-hand coordinate system defines the positive loading directions as, X—anterior, Y—lateral, Z—superior. Rotations are defined positive in the direction the fingers curl when “holding” the axis of interest and pointing the thumb in the positive direction. The two harvest sites used in this study is indicated with dashed lines

To further evaluate the stability of an osteotomy for different implant configurations (*i.e.,* entry points, directions, and implant lengths), virtual models of mechanical systems can be used. The finite element (FE) method is a mathematical model to numerically solve partial differential equations that describe mechanical systems in fields such as structural analysis, heat transfer, and fluid flow [16]. When the FE method is used to model mechanical systems, the study geometry is discretized into smaller, simpler parts called finite elements, where each element is associated with a material model that describes its mechanical behavior. To complete the mathematical model, boundary conditions (e.g., supports, connections with surrounding structures) and external loads (e.g., force, pressure, prescribed motions) are applied (see Fig. 1). If the structure consists of several interacting parts, a contact algorithm can be applied preventing the parts to penetrate each other. FE analysis to simulate stress, strain, and stiffness within orthopedics has become increasingly popular [17, 18]. Vafaeian et al*.* performed an extensive review on FE analysis of acetabulum pressure related to surgery of developmental dysplasia of the hip [19]. Zou et al. used FE analysis to optimize the position of the acetabulum during a virtual PAO [20]. In addition, FE analysis has been used to study the influence of fixation parameters on biomechanical stability, e.g., in a proximal femoral osteotomy [21], sacroiliac joint fixations [22], or treatment of supracondylar humerus fractures [23]. Though virtual models can never completely replace clinical trials or ex vivo models like that of Yassir et al., they enable paired testing using the exact same “virtual” object and, as such, can be used to evaluate the stability of implants for different implant configurations.

In this study, we aim to evaluate how different implant configurations affect the stability of the iliac osteotomy in a TPO using finite element analysis with the purpose to explore if bioabsorbable screws can enable favorable biomechanical implant constructs that cannot be utilized using metal screws or K-wires.

## Materials and methods

A base FE model of an average shaped hemi-pelvis [24], consisting of the left ilium, the acetabulum, and a wedge-shaped graft (see description below) was created for simulations in Abaqus/Standard 2020.HF3 (Dassault systemes, Vélizy-Villacoublay, France). For information about the mathematical implementation to solve the FE equations, we refer to the Abaqus theory manual.^1^ To illustrate and define loading directions for the current study, the pelvis was positioned in the standing anatomical position [25], see Fig. 1. The FE model consists of 46 000 tetrahedral solids (Abaqus element formulation C3D10), representing trabecular bone and 10 000 triangular shell elements (Abaqus element formulation STRI65), representing cortical bone, for an average element size of 3.2 mm for the solids and 2.9 mm for the shells. The cortical and trabecular bones were modeled using an isotropic linear elastic material model, with Young’s modulus 17 000 MPa and 70 MPa, respectively [26]. For the cortical bone, a uniform thickness of 1 mm was assumed [26].

To simulate a triple osteotomy as described by Carlioz [3], a virtual osteotomy was performed in the base geometry prior to meshing, superior to the acetabulum along with cuts in the ischial and pubic bones as illustrated in Fig. 1. The re-alignment of the acetabular fragment simulated a standardized correction with + 15 deg Y + rotation and 5 deg X- rotation. The traditional harvest site at the superior anterior iliac spine and the more posterior alternative harvest site are visualized in Fig. 1. For the model, a standardized graft was harvested from the iliac wing (graft harvest site B, Fig. 1) and placed centrally in the osteotomy plane. This standardized graft was used for all models (even when entry points were from harvest site A). No elements were removed from the harvest site assuming that the stability of the osteotomy and the acetabular fragment would be unaffected by the removal of these elements. The acetabular fragment was not moved in the X-axis, even though a displacement in X+ is mandated by some surgeons.

The implants were modeled using beam elements with a circular cross section of 4.5 mm in diameter and using an isotropic linear elastic material model with a Young’s modulus of 5 000 MPa.^2^ All nodes that belonged to the ilium, the graft, and the acetabulum, enclosed by the cylinder representing the 4.5-mm-diameter screw, were fully constrained (Abaqus *Tie) to the implant beam elements. These nodes are highlighted as white dots in Fig. 1. This simulates a fully threaded screw, and as such, no beam pretension was applied. This means that the contact stress was zero at the start of each simulation.

To model the boundary conditions, the ilium was constrained in all translational and rotational DOF at the SI joint surface, see Fig. 1, while the distal fragment was considered un-constrained. Contacts (Abaqus *Contact Pair, Surafce_To_Surface, with friction coefficient 0.2), as suggested^3^ to be used together with element type C3D10, were defined between ilium and the graft as well as the acetabulum and the graft.

Pelvis loading was applied at the center of the femoral head, for one direction (X/Y/Z translation or X/Y/Z rotation) at a time, using prescribed unit displacements. The load, 1 mm translation or 1 degree rotation, was distributed over the lunate surface of acetabulum (using Abaqus kinematic *COUPLING, degrees of freedom (DOF) 1–6).

Finally, to be easily comparable to the work by Yassir et al., stability of the virtual osteotomy was evaluated in terms of mechanical flexibility^4^ (i.e., displacement for a given load measured in [mm/N]), where higher stability corresponds to lower flexibility. For each implant configuration, the relative flexibility from translational loads was evaluated in all DOFs except for Z-, describing a clinically unlikely distracting force on the osteotomy. Relative flexibility from rotational loads was calculated for all DOFs. Relative, in this context, means that the average flexibility was computed for all implant configurations, and then, each configuration was normalized by this average. This was performed separately for each direction and DOF. Since the load was applied at the center of acetabulum, the lever arm caused any translation in X and Y to be coupled by rotation. For example, a rotation around Y + will correspond to a translation in X-. A rotation around the Z-axis, however, does not couple with a translation since no translation include a lever arm on this axis.

For the present simulation, five implant placement configurations, corresponding to five different FE models, were chosen to represent different clinically feasible fixation solutions of the osteotomy (Table 1). In configurations A, B, and C, the entry points were chosen to enable later implant removal, using the classic harvest site of the graft from the iliac crest (harvest site A, Fig. 1). D and E were configured to include surgically feasible entry points that did not need to be easily accessible for later extraction. These entry points included the inner and outer aspects of the iliac wing, as well as entry points from a more proximal harvest site (Fig. 1, harvest site B and Table 1, models D and E). The principles behind the implant configurations for respective model are presented in Table 1. For all chosen placement configurations, three principles were adhered to: (I) Two out of three screws should pass through the triangular graft, (II) the screws should, if not otherwise stated, not end superior to the acetabulum, and (III) the distance between the implants in the graft plane should be maximized, considering surgical constraints and I and II.

**Table 1:**
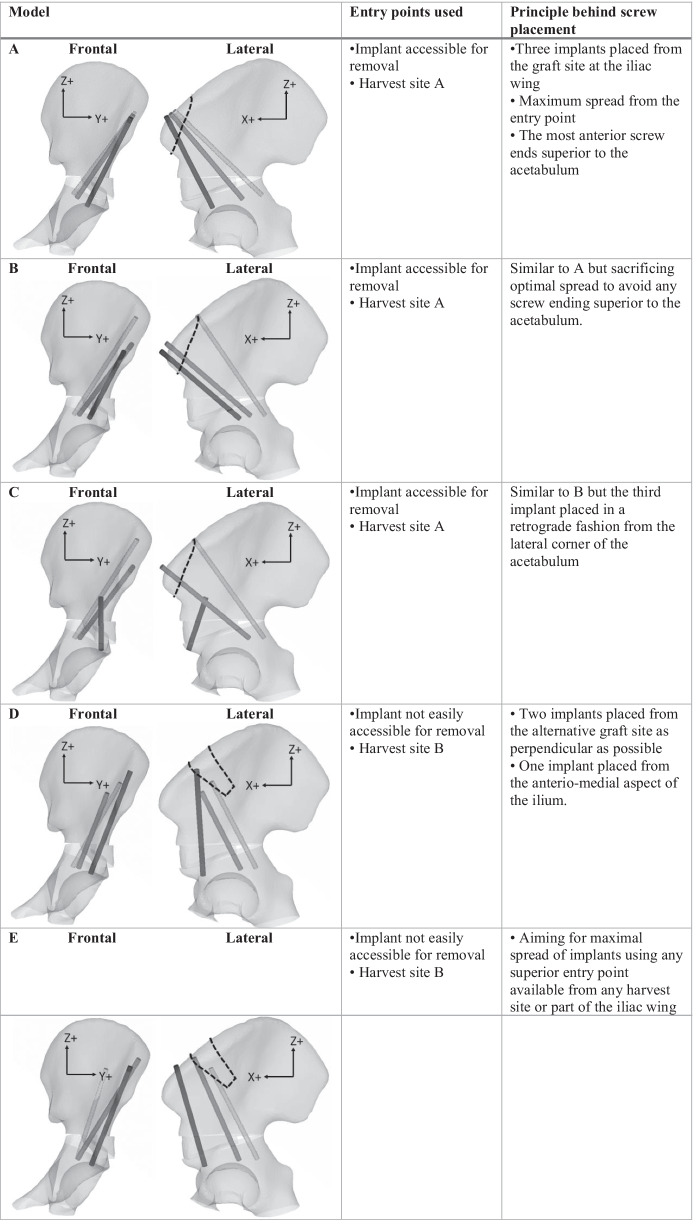
Description of implant placements for the five FE models. The model is of the left ilium and acetabulum. The frontal view shows the pelvis and implants in a posterior view, and the lateral in a medial view. The harvest site is indicated with dashed lines

## Results

**Fig. 2:**
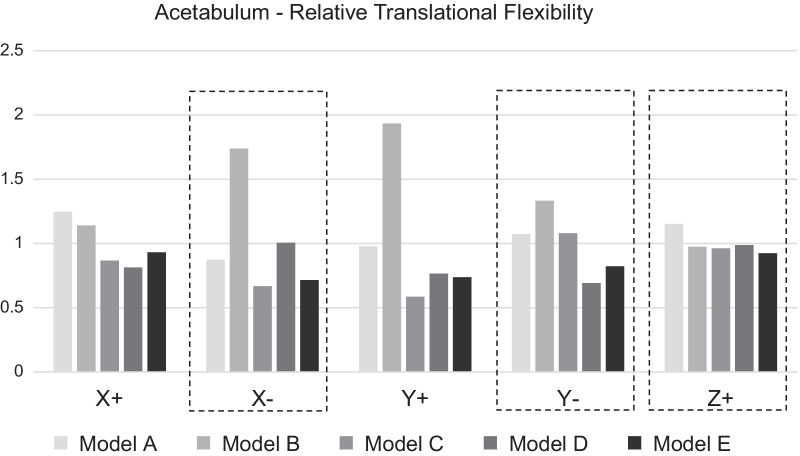
The flexibility for translational loads normalized to the average flexibility for each load direction, meaning that lower bars represent greater stability for the respective direction

Figure 2 shows the resulting flexibility for different implant placements. The most relevant results from a clinical point of view (X−, Y−, Z+) are highlighted with dashed boxes. The values for X+ (lateralization of the distal fragment) and Y+ (anterior displacement of the distal fragment) are presented for completeness but usually do not present a problem in clinical practice. Tests for Z- were, as mentioned before, omitted due to distraction never being a relevant factor in this case. The flexibility for rotational loads behaved, like expected, in correspondence with the translational loads, and the results for rotation in X+ mirrored the translation in Y+ almost exactly. The complete results for rotational loads are available in Additional file 1: Appendix 1.

Averaging the relative translational flexibility in Fig. 2, over the three relevant directions (Z+, X−, and Y−), results in average flexibilities from 0.82 for model E to 1.35 for model B (model A = 1.03, Model C and D = 0.90), meaning that model E is on average the least flexible and model B the most flexible. To add some granularity, model E showed the most consistent low flexibility of all models with 18% less flexibility compared to the average flexibility. Model A shows lower flexibility than model B in the clinically relevant X-, Y-directions (however, both have 3% and 35% higher flexibility compared to the average) and roughly equivalent in Z+. Model C, with a screw placed from the distal fragment, was equivalent or less flexible in all degrees of freedom compared to A and B and also performed on par with D and E in all dimensions except Y− where C showed more flexibility. It can also be noted that D, with a flexibility comparable to C and E in most dimensions, has a markedly higher flexibility in X−. The Z+ movement, which largely corresponds to a closing of the osteotomy, exhibited similar flexibility for all implant positions.

## Discussion

The FE method has been proven to be useful for postoperative modeling of different orthopedic applications and has been used to evaluate different fixation methods for PAO [20, 27, 28]. Despite TPO being a mainstay of advanced surgical treatment of multiple conditions, FE analysis has, to our knowledge, not previously been applied to evaluate the stability of different implant constructs.

In this study, we present a FE analysis of how screw placements affect the stability of a standardized TPO. Model E, with the most entry points available, clearly had the best results from a stability point of view. Model C, with a distally placed screw, also showed high stability, but placing a screw from the acetabular fragment has drawbacks. Firstly, extraction of a screw (or pin) from this entry point can be challenging, secondly the antero-lateral corner superior to the acetabulum is small and can potentially break, and thirdly implants in this location may also interfere with hip abduction. In the other models with all screws placed from the superior fragment (A, B and D), the option to place the screws from more entry points (D) outperformed the models with less entry points available (A and B).

Adamczyk et al. used an in vitro model to compare the stability of bioabsorbable (PLLA^5^) and metal screws after a TPO [29] in weight-bearing conditions. No significant difference was found at physiological loads, when comparing the two materials in both the standing and spica cast positions. This was likely, according to the authors, due to the inherent stability of the remaining intact pelvic ring, as compression of the osteotomy will cause bony contact that carry the load regardless of implant material. This result is in line with our findings for Z+ loading, where implant position had a limited effect on stability when compressing the osteotomy. Hence, it can be suspected that the osteotomy and graft position are more important than the strictly mechanical properties of the implant when studying a weight-bearing scenario. For loading directions where the implants are mainly subjected to bending forces, e.g., in the X- and Y-directions, the resulting stability will be a trade-off between strong material properties and optimal placement. Bioabsorbable screws enable a superior implant placement which should be considered along with other benefits, like not needing extraction and less risk of implant migration, when making a judgment for the best procedure. Our present findings show, in line with two basic orthopedic principles, that implants should be spread out in all dimensions over the osteotomy and that implant placements more perpendicular to an osteotomy improve stability. Both principles are easier to adhere to when more entry points are available. Bioabsorbable screws are not as strong as an equivalent metal screw, but the benefit of more placement options may, to a certain degree, compensate this limitation. A TPO can, however, also be performed on adults, and in these cases, extraction of metal screws may not be necessary and the benefit and indication of bioabsorbable implants thus decrease.

In the present study, we have not used physiological loads but rather focused on a generic prescribed displacement of 1 mm or 1 degree in a given direction. The displacements for different fixation methods are in the same order of magnitude as found by Yassir et al. [11] (< 2 mm displacement and < 3 deg rotation), supporting the load used in the present study.

A load analysis based on everyday motions or standing loads might have modified the results [30], but this was out of the scope of the study. Since the TPO is normally followed either by a hip spica, an orthosis or non-weight-bearing in a wheelchair, physiological loads in a standing position are of less relevance until bony healing has occurred (6–8 weeks postoperatively). The present study, therefore, focused on these, crucial, first weeks postoperatively rather than stability during a later stage.

A validation of the FE model by comparing the model predictions to a physical test was not feasible in the current study, since no physical experiment measuring stiffness in all DOFs exists in the literature. The only relevant study with a similar test setup, performed on cadaver specimens, is Yassir et al. [15] enabling a plausibility check of the model predictions in vertical loading (Z+ in the current study). In the study by Yassir et al., the authors report a 0.7 mm deformation for a screw configuration similar to present Model C, and a 1.2 mm deformation for a screw configuration similar to present Model A/B with vertical loading (Z+ in the current study), for an external load of 450 N (equivalent to 225 N per hip joint). The Z + stiffness in the current study ranged from 626 to 748 N/mm (see Additional file 1: Appendix) for Models A-C, corresponding to a 0.30–0.36 mm deformation. This is slightly smaller to the deformations measured by Yasser et al., but it should be pointed out that the FE model used in this study represents a “perfect” osteotomy, with all bony parts perfectly cut and aligned. This is not the case in reality, where any imperfections will result in larger deformation. In addition, Yasser et al. point out the risk of “increased osteotomy fragment motion secondary to screw loosening in the later trials,” regarding the repeated testing. In the light of this, the model predictions seem to be plausible, at least for vertical loading in the Z + direction.

In the present model, the influence of muscle forces and soft tissues was also discarded. Muscles and soft tissues can provide stability to the pelvis, while on the other hand, muscle forces can cause greater loading [31]. Due to the partial immobilization of the patient following a triple pelvic osteotomy, the variation in loading from muscles forces is considered to be of less importance.

All displacements are not of equal clinical relevance, and a distracting force on the osteotomy is, for example, irrelevant. In clinical practice, it is widely known that the movements normally are associated with instability and a collapse of an osteotomy is firstly a “closing” of the osteotomy (mostly Z+ movement in our model) and/or a postero-medial dislocation of the distal fragment (X− in our model). A rotation around the X-axis affects the ante/retroversion of the acetabulum with both being undesirable but an increased retroversion even less so. A displacement in both X− and Y− negates the correction achieved and means that the distal fragment may lose the hinge of cortical bone in the infero-medial corner of the osteotomy.

Due to the complexity of the three-dimensional shape of the pelvis, the theoretically possible entry points and subsequent angles and lengths of an implant are innumerable and the modeling had to be restricted to study only a few clinically feasible solutions. Our study does not present reference values but intend to serve as a guide for surgical planning of TPO by outlining factors affecting stability and support. Moreover, our study was performed with a singular type of implant, corresponding to a 4.5-mm resorbable 85L/15G PLGA screw. Compared to commonly used 2-mm-steel K-wires, the 4.5-mm bioabsorbable screw has 60% less bending stiffness. Thus, for loading directions where the implants are mainly subjected to bending forces, e.g., in the X- and Y-directions, the joint overall flexibility should be comparable. For loading directions where the implants are mainly subjected to axial loads, (Z-), a 2-mm K-wire is almost 10 times stiffer than a 4.5-mm bioabsorbable screw, but this is of less clinical relevance. Also, lacking threads, the K-wire cannot fully utilize its axial stiffness, so the pull-out strength of a 4.5-mm bioabsorbable screw remains superior. Depending on the specific type of implant, the absolute magnitude of the results will vary to some extent, but the relative stability/flexibility between different implant configurations is expected to be consistent.

Although the study demonstrated the comparison of different fixation systems, the modeling had its limitations. Similar to other established finite element models of the pelvis [28], the implant screws were simplified as homogeneous with isotropic materials. Furthermore, a constant cortical thickness and homogeneous and isotropic material properties were assigned to the pelvis bone. This is a simplification compared to some other models in the literature that include a non-homogeneous distribution of trabecular bone properties [28, 32, 33]. However, since the aim of this study was to compare the biomechanical performance of different implant configurations in identical conditions, and not to study the stress–strain state of the bone or the contact stress of the osteotomy, this simplification was justified. This is further motivated by analyzing differences of relative flexibility, as a small error in structural stiffness would be treated equally for all configurations by the normalization of the results.

Using a similar motivation, we have not performed any mesh converges study. Since flexibility (or stiffness) relates to the displacement while stress/strain relates to the derivative of the displacement, structural flexibility will converge much faster than stress/strain. Therefore, mesh convergence studies are of greater importance for stress or strain analyses. A relevant example is presented in the ABAQUS (2016) user guideline, where even a very coarse mesh consisting of just 14 elements gives a stiffness prediction within 3% of the accurate result, while the stress at the critical area is not converged even using the finest mesh consisting of 1800 elements.

Moreover, the individual shape of the pelvis may vary greatly, affected by sex, age, and as shaped by the underlying condition necessitating surgery. The shape of the pelvis should not, however, greatly affect the relative stability between the screw configurations. Nevertheless, additional studies are warranted to evaluate the applicability of our findings in clinical practice. While virtual models cannot completely replace experimental testing, they provide a valuable tool that is rapidly evolving.

## Conclusion

In a FE model of a TPO, different implant placement configurations had a substantial impact on the stability of the osteotomy. As predicted, a greater spread of the implants over the osteotomy and configurations more perpendicular to the osteotomy improved the stability. The findings suggest that bioabsorbable screws in TPO, enabling a higher degree of freedom when selecting entry points, provide superior preoperative stability as compared to TPO with only traditional entry points.

## Supplementary Information

**Additional file 1: Appendix 1.** Tabulation of all results from the FE simulations.

## Data Availability

All data generated or analyzed during this study is included in the published article, including Additional file 1: Appendix.

## Abbreviations

FE: Finite element
MPa: Mega Pascal
PAO: Periacetabular osteotomy
PLGA: Poly-lactic-co-glycolic acid
PLLA: Poly-(L)-lactic acid
SO: Salter osteotomy
TPO: Triple pelvic osteotomy

## Footnotes

1 ABAQUS (2016) Theory Manual. Version 2016, Dassault Systemes Simulia, Inc.

2 Reported characteristics of a 4.5-mm 85L/15G Poly lactic-co-glycolic acid (PLGA) cannulated screw, previously used for pelvic osteotomies, by the manufacturer of Activa screw (Bioretec, Tampere, Finland).

3 ABAQUS (2016) Getting Started with Abaqus/CAE. Version 2016, Dassault Systemes Simulia, Inc.

4 3 Flexibility is the inverse of stiffness, where stiffness is defined as resistance to deformation in response to applied force and measured in [N/mm]).

5 Poly-(L)-lactic acid. A polymer comparable, but not identical, to 85L/15G PLGA screws used in this article.
