# Supplementary material for: Postoperative stability following a triple pelvic osteotomy is affected by implant configuration: a finite element analysis
Source: J Orthop Surg Res. 2022 May 15;17:275. doi: 10.1186/s13018-022-03169-3 (PMC9107681; doi:10.1186/s13018-022-03169-3)
Supplement: Supplementary file 1 — Additional file 1: Appendix 1. Tabulation of all results from the FE simulations. [file 13018_2022_3169_MOESM1_ESM.docx]

Appendix 1 – Flexibility for rotational loads

Figure A- 1. The flexibility for **rotational** loads normalized to the average flexibility for each load direction, meaning that lower bars represent greater stability for the respective direction.

Appendix 2 – Tabulation of all results

In Table B- 1 the results from the FE simulations using translational unit displacement are presented. For each load case a unit displacement of 1 mm was applied. The result in each cell corresponds to the force that were required to pull the femur head center 1 mm in that direction.

Table B- 1. Results from FE simulations using translational displacement unit loads presented in stiffness form

| Direction | **X+** | **X-** | **Y+** | **Y-** | **Z+** |
| --- | --- | --- | --- | --- | --- |
| Unit | [N/mm] | [N/mm] | [N/mm] | [N/mm] | [N/mm] |
| Model A | 38.4 | 36.3 | 9.1 | 8.6 | 625.8 |
| Model B | 42.0 | 18.3 | 4.6 | 6.9 | 739.9 |
| Model C | 55.2 | 47.6 | 15.2 | 8.5 | 748.4 |
| Model D | 58.9 | 31.6 | 11.6 | 13.3 | 729.5 |
| Model E | 51.4 | 44.4 | 12.1 | 11.2 | 778.7 |

In Table B- 2 the results have been recalculated to flexibility form by taking the inverse of each cell value. The last row represents the average flexibility for each column (each load direction)

Table B- 2. Results from FE simulations using translational displacement unit loads presented in flexibility form

| Direction | **X+** | **X-** | **Y+** | **Y-** | **Z+** |
| --- | --- | --- | --- | --- | --- |
| Unit | [mm/N] | [mm/N] | [mm/N] | [mm/N] | [mm/N] |
| Model A | 0.0261 | 0.0275 | 0.1098 | 0.1163 | 0.00160 |
| Model B | 0.0238 | 0.0547 | 0.2171 | 0.1446 | 0.00135 |
| Model C | 0.0181 | 0.0210 | 0.0657 | 0.1171 | 0.00134 |
| Model D | 0.0170 | 0.0317 | 0.0860 | 0.0750 | 0.00137 |
| Model E | 0.0194 | 0.0225 | 0.0828 | 0.0891 | 0.00128 |
| Average | 0.0209 | 0.0315 | 0.1123 | 0.1084 | 0.00139 |

In Table B- 3 the results in each cell in Table B- 2 have been normalized (divided by) the average value last in each column in Table B- 2. These are the results presented in the main part of the paper.

Table B- 3. Results from FE simulations using translational displacement unit loads presented in normalized flexibility form

| Direction | **X+** | **X-** | **Y+** | **Y-** | **Z+** |
| --- | --- | --- | --- | --- | --- |
| Unit | [-] | [-] | [-] | [-] | [-] |
| Model A | 1.25 | 0.87 | 0.98 | 1.07 | 1.15 |
| Model B | 1.14 | 1.74 | 1.93 | 1.33 | 0.97 |
| Model C | 0.87 | 0.67 | 0.59 | 1.08 | 0.96 |
| Model D | 0.81 | 1.01 | 0.77 | 0.69 | 0.99 |
| Model E | 0.93 | 0.72 | 0.74 | 0.82 | 0.93 |

In Table B- 4 the results from the FE simulations using rotational unit displacement are presented. For each load case a unit displacement of 1 degree rotation was applied. The result in each cell corresponds to the moment that were required to pull the femur head center 1 degree in that direction.

Table B- 4. Results from FE simulations using rotational displacement unit loads presented in stiffness form

| Direction | **X+** | **X-** | **Y+** | **Y-** | **Z+** | **Z+** |
| --- | --- | --- | --- | --- | --- | --- |
| Unit | [Nmm/deg] | [Nmm/deg] | [Nmm/deg] | [Nmm/deg] | [Nmm/deg] | [Nmm/deg] |
| Model A | 358.7 | 263.6 | 1377.0 | 2134.4 | 744.5 | 1220.1 |
| Model B | 179.5 | 252.0 | 612.1 | 2490.8 | 593.2 | 625.4 |
| Model C | 555.1 | 269.4 | 2566.1 | 2664.2 | 1145.7 | 1360.2 |
| Model D | 465.0 | 397.0 | 1321.3 | 3210.3 | 676.0 | 1339.8 |
| Model E | 478.7 | 338.6 | 1878.2 | 2993.5 | 860.1 | 1328.1 |

In Table B- 5 the results have been recalculated to flexibility form by taking the inverse of each cell value. The last row represents the average flexibility for each column (each load direction)

Table B- 5. Results from FE simulations using rotational displacement unit loads presented in flexibility form

| Direction | **X+** | **X-** | **Y+** | **Y-** | **Z+** | **Z-** |
| --- | --- | --- | --- | --- | --- | --- |
| Unit | [deg/Nmm] | [deg/Nmm] | [deg/Nmm] | [deg/Nmm] | [deg/Nmm] | [deg/Nmm] |
| Model A | 0.00279 | 0.00379 | 0.00073 | 0.00047 | 0.00134 | 0.00082 |
| Model B | 0.00557 | 0.00397 | 0.00163 | 0.00040 | 0.00169 | 0.00160 |
| Model C | 0.00180 | 0.00371 | 0.00039 | 0.00038 | 0.00087 | 0.00074 |
| Model D | 0.00215 | 0.00252 | 0.00076 | 0.00031 | 0.00148 | 0.00075 |
| Model E | 0.00209 | 0.00295 | 0.00053 | 0.00033 | 0.00116 | 0.00075 |
| Average | 0.00288 | 0.00339 | 0.00081 | 0.00038 | 0.00131 | 0.00093 |

In Table B- 6 the results in each cell in Table B- 5 have been normalized (divided by) the average value last in each column in Table B- 5. These are the results presented in Appendix 1.

Table B- 6. Results from FE simulations using rotational displacement unit loads presented in normalized flexibility form

| Direction | **X+** | **X-** | **Y+** | **Y-** | **Z+** | **Z-** |
| --- | --- | --- | --- | --- | --- | --- |
| Unit | [-] | [-] | [-] | [-] | [-] | [-] |
| Model A | 0.97 | 1.12 | 0.90 | 1.24 | 1.03 | 0.88 |
| Model B | 1.93 | 1.17 | 2.02 | 1.06 | 1.29 | 1.72 |
| Model C | 0.63 | 1.10 | 0.48 | 0.99 | 0.67 | 0.79 |
| Model D | 0.75 | 0.74 | 0.94 | 0.82 | 1.13 | 0.80 |
| Model E | 0.73 | 0.87 | 0.66 | 0.88 | 0.89 | 0.81 |
